# Evaluation of Traditional Chinese Medicinal Plants for Anti-MRSA Activity with Reference to the Treatment Record of Infectious Diseases

**DOI:** 10.3390/molecules17032955

**Published:** 2012-03-09

**Authors:** Guo-Ying Zuo, Xin-Juan Zhang, Cui-Xian Yang, Jun Han, Gen-Chun Wang, Zhong-Qi Bian

**Affiliations:** 1 Research Center for Natural Medicines, Kunming General Hospital, PLA, Kunming 650032, China; Email: shmily_zxj2008@163.com (X.-J.Z.); ycx484@126.com (C.-X.Y.); kmwgc12@126.com (G.-C.W.); 2 Kunming Medical College, Kunming 650032, China; 3 Yunnan Traditional Chinese Medical College, Kunming 650500, China; Email: hanzjn@126.com; 4 Center for Infectious Diseases, Kunming General Hospital, PLA, Kunming 650032, China

**Keywords:** Chinese medicinal plants, traditional Chinese medicine (TCM), methicillin-resistant *Staphylococcus aureus* (MRSA), inhibition zone diameters (IZD), minimal bactericidal concentrations (MIC)

## Abstract

The *in vitro* antimicrobial activities of 30 Chinese medicinal plants were evaluated with reference to the treatment record of infectious diseases in the Traditional Chinese Medicine (TCM) literature. The plant materials were extracted with 80% ethanol and the extracts were primarily screened against conventional clinical pathogens like *Staphylococcus aureus*, *Escherichia coli*, *Pseudomonas aeruginosa *and *Candida albicans* by the agar diffusion method. Their inhibition zone diameters (IZDs, mm, 50 mg/mL) ranged from <8 to 24. The 21 extracts which showed IZDs ≥10 mm against MSSA were also active against methicillin-resistant *S*. *aureus *(MRSA) with lower IZDs of 9.0–18.8 mm. They were further subjected to minimal inhibitory concentration and minimal bactericidal concentration (MIC/MBC, μG/mL) assays, which were 8-2,048/32->2,048 by the standard broth microdilution method. The seven extracts from *M*. *yunnanensis*, *S*. *sinensis*, *G*. *morella*, *E*. *daneillii*, *M*. *squamulata*, *S*. *arborescens* and *B*. *hancei *were determined as the most active extracts, with MICs of 8–64 μg/mL. The results were in good agreement with their traditional applications in skin and other infections.

## 1. Introduction

*Staphylococcus aureus* infections range from common skin infections, such as furunculosis and impetigo, to severe deep-seated infections. *S*. *aureus* ranks first or second among bacterial pathogens causing bloodstream infections. It is the leading cause of nosocomial pneumonia and it also causes infections of surgical wounds and prosthetic implants. Clinical isolates of methicillin-resistant *Staphylococcus aureus *(MRSA) have become the most common cause of infections among pathogenic bacteria around the Globe, and many life-threatening diseases such as endocarditis, pneumonia and toxin shock syndrome are ascribed to them. Contrary to methicillin-susceptible *S*. *aureus *(MSSA), MRSA tend to be multi-drug resistant (MDR), that is, resistant not only to β-lactam antibiotics but also to a wide range of different antibiotic classes, such as fluoroquinolones, tetracyclines, macrolides, lincosamides and aminoglycosides, and even strains of vancomycin intermediate susceptible or full resistant (VISA and VRSA, respectively) have emerged [1]. Therefore, the search for novel anti-MRSA agents is urgently needed. Meanwhile, great emphasis has been placed on the value of plants used in ethnomedicine and traditional medicine for drug discovery has currently been laid greater stress worldwide [2,3,4,5].

Traditional Chinese medicines (TCM), like Ayurveda, Unani and Kampo, have flourished as systems of medicine in use for thousands of years [2]. With more than 5,000 years of Chinese history and as a part of Chinese culture, TCM mainly used plant materials in organized traditional medical systems which have been playing a critical role in fighting various diseases and maintaing human health. Thousands of Chinese herbal medicines have been recorded in a great variety of national and local literatures such as *Shen Nong*’*s Materia* *Medica* [6], *Compendium of Materia Medica* [7], *Dictionary of Chinese Materia Medica * [8], *Chinese Materia Medica* [9], *Compilation of Chinese Herbal Medicine* [10] and *Selected Yunnan Traditional Chinese Herbs* [11] from ancient to modern times. They have been widely used throughout the country or in local areas by the Chinese people of all nationalities.

In TCM clinical practice, the Chinese disease name “*Chuang-Yang*” (pyogenic infection and ulceration of skin) is a general terminology for the surgical and skin disease visible over the body surface and dermatosis, including “*Yong Ju*, *Ding-Chuang*, *Jie-Zhong*, *Liu-Zhu*, *Liu-Tan*, *etc.*” (carbuncle, deep-seated sore, furuncle, multiple abscess, tuberculosis of bone and joint, scrofula, *etc*.). The diseases are commonly seen in clinic, due to stagnation of *qi* and blood stasized from the attack of *evils* and consumption of blood by *heat-evil* [12].

Many Chinese herbal medicines (or medicinal plants) have been documented as treatment of these diseases, which prompted us to investigate their inhibitory activity against MRSA. We herein report the *in vitro* anti-MRSA effects of a collection of 30 Chinese medicinal plants with reference to the treatment record of skin and other kinds of infections (Table 1).

**Table 1:** Traditional indications and phytohemical constitution of the 30 Chinese plants.

No.	Species	Traditional Indication	Phytohemical Constitution
1	*A*. *nepalensis*	bleeding of the nose, enteritis and dysentery	tannins, triterpenoids, flavonoids, phenols
2	*B*. *balsamifer*	anti-rheumatism, ringworm and sores, dysentery, detoxification and snake bite	flavonoids, simple terpenoids
3	*B*. *hancei*	heat clearing and detoxicating, dysentery, jaundice, boils, swelling, tuberculosis injury hematemesis, osteomyelitis, periostitis, rheumatism and pain	hydroxytyrosol derivatives and glycosides
4	*C*. *austroglauca*	astringing sores, carbuncles, dysentery, hemostasis and vaginal discharge	none
5	*C*. *japonica*	carbuncles, boils, mumps, erysipelas, rheumatism, jaundice, dysentery, hematuria and gonorrhea	flavonoids (delphinidin glycosides)
6	*C*. *orbiculatus*	dysentery, multiple abscess, Herpes zoster, detoxification, inflammatory, cellulites and snake bite	sesquiterpene, flavonoids
7	*C*. *orchioides*	diarrhea, ulcer, pus and muscles atrophy	triterpenoids, lignans, flavonoids, alkaloids, stereoids
8	*C*. *prainii*	antipyresis, diuretic and chyluria	alkaloids, polyphenols, flavonoids
9	*E*. *Burm*	heat clearing and detoxicating, pharyngitis, dysentery, diarrhea, furuncle ulcer, skin itching, swelling and pain of hemorrhoids, throat red and swollen, bleeding gums and traumatic injury.	quinones, triterpenoids, flavonoids
10	*E*. *daneillii*	diarrhea, abdominal pain and vomiting	alkaloids, flavonoid glycosides, flavaprin, limonoids
11	*E*. *fortunei*	chronic diarrhea, dysentery, dispersing blood stasis and traumatic bleeding	alkaloids, triterpenoids, flavonoids
12	*E*. *laxiflorus*	pesticide, rheumatism, bone fractures and hemoptysis	alkaloids, triterpenoids, flavonoids, sesquiterpenes (agarofurans)
13	*G*. *morella*	wound rot, carbuncle, tinea, ulcer and sore, anthelminthic and containing toxic substances	phenols (gambogic acid), flavonoids (xanthones), triterpenoids
14	*I*. *simonsii*	scabies, bladder hernias, mixed cropping of edible spices and containing toxic substances	terpenoids, lignans, flavonoids, phenols
15	*K*. *angustifolia*	anti-infection, swelling and pain, ulcer and enteritis and heat stroke	lignans, triterpenoids
16	*L*. *lancifolia*	stomach pain, vomiting and swelling	alkaloids, terpenoids, flavonoids, essential oils
17	*M*. *hongheensis*	vomiting, diarrhea, dysentery, constipation and geriatric hacking cough	alkaloids
18	*M*. *salicina*	carbuncle, furunculosis and sore pain	alkaloids, lignans
19	*M*. *squamulata*	scabies, carbuncle boils swollen poison, hemorrhoids, enterobiasis, beriberi, rheumatoid and snake bite	triterpenoids
20	*M*. *yunnanensis*	hepatitis, sore, otitis media, stomach and duodenal ulcer, enlarged spleen and boils swelling, hematuria leucorrhea and traumatic bleeding	Polyphenols,tannins，flavonoids,coumarins, various terpenoids
21	*O*. *javanica*	lump in the abdomen, boils and swelling of throat and containing toxic substances	falcarindiol, carotatoxin, 5-allylpyrogallol
22	*P*. *edulis*	(no record)	unsaturated organic acids
23	*P*. *molle*	carbuncle, swollen abscess, fistula and scrofula	tannins, flavonoids, alkaloids
24	*R*. *japonicus*	subsiding swelling, jaundice, malaria lymph node tuberculosis and pterygium	lactones (anemonin, protoanemonin), flavonoids
25	*S*. *arborescens*	HBV (skimmianine), rheumatoid, paralysisa, beriberi, and containing toxic substances	alkaloids, coumarins, triterpenoids, phenols
26	*S*. *davidii*	antipyretic, detoxicate, subsiding swelling, laryngitis, pneumonia, dysentery, cystitis and edema	polyphenols
27	*S*. *parasitica*	anti-rheumatism and anticancer	flavonoids
28	*S*. *sinensis*	furuncle and swelling	benzoquinone, tannins, phenols, lignans, flavonoids, triterpenoids
29	*S*. *tamariscina*	inflammation, pharyngolaryngitis and bacteriostasis	flavonoids, phenol glycosides, trehalose
30	*S*. *viridis*	urticaria, herpes zoster, rheumatism and analgesia	lignans, triterpenoids, organic acids

## 2. Results and Discussion

### 2.1. Results

Table 1 shows the recorded TCM indications and phytohemical constitution of the 30 Chinese medicinal plants [6,7,8,9,10,11]. The antibacterial susceptible spectrum of the tested seven MRSA strains is listed in Table 2. The initial screening results of the 30 plant extracts against MSSA and other conventional standard strains of *Escherichia coli* (ATCC 25922), *Pseudomonas aeruginosa *(ATCC 27853) and *Candida albicans *(ATCC Y0109) are expressed as inhibition zone diameters (IZDs) in Table 3, while anti-MRSA activities of the selected 21 extracts which were active with IZDs ≥10 mm against MSSA were shown in Table 4. Their corresponding MICs/MBCs (µg/mL) are shown in Table 5.

**Table 2:** The antibacterial agent-susceptibility test results of MRSA strains.

MRSA Strains	Resistant (R)	Intermediate (I)	Susceptible (S)
MRSA 008	PEN, OXS, AMP, LEV, CTX, PIP/S, ERY, CLI, AZM, RIF	NONE	VAN, TEC, LNZ, FOS,
MRSA 082	AMP, CLI, RIF, CTX V, CIP, LEV, CAZ	FOS	LNZ, VAN
MRSA 092	AZM, OXS, AMP, FOX, CLI, CLR, LEV	NONE	COT, RIF, PIP/S, VAN, GAT, FOS
MRSA 123	PEN, OXS, FOX, IPM CTS	NONE	LNZ, VAN, MXF, FOS
MRSA 144	PEN, AMP, OXS, FOX, FUR, CFZ, AZM, RIF, CLI, CLR	NONE	VAN, FOS
MRSA 189	PEN, OXS, AMP, FOX, FUR, CTX, COT, CIP, LEV	FOS	VAN, LNZ, MXF
MRSA 321	CLR, CLI, AZM, PEN, OXS, AMP, ERY. CTX, FUR, LEV	CAT	VAN, GAT, PIP/T

AMP: ampicillin; AZM: azithromycin; CAT: cefathiamidine; CAZ: ceftazidime; CFZ: cefazolin; CIP: ciprofloxacin; CLI: clindamycin; CLR: clarithromycin; COT: Cotrimoxazole; CTS: cilastatin sodium; CTX: Cefotaxime; ERY: erythromycin; FOS: fosfomycin; FOX: cefoxitin; FUR: cefuroxime; GAT: gatifloxacin; IPM: imipenem; LEV: levofloxacin; LNZ: linezolid; MXF: moxifloxacin; OXS: oxacillin; PEN: Penicillin; PIP: piperacillin; PIP/S: piperacillin/sulbactam; PIP/T: piperacillin/tazobactam; RIF: rifampin; TEC: teicoplanin; VAN: Vancomycin.

**Table 3:** Screening results of the antimicrobial activities of the extracts from 30 Chinese medicinal plants (IZD: mm) ^a^.

No.	Species	Part ^b^	Weight (g) ^c^	Ratio (%) ^d^	SA ^e^	EC ^f^	PA ^g^	CA ^h^
1	*A*. *nepalensis*	TBL	3.85	7.7	14	<8	12	9
2	*B*. *balsamifer*	WP	0.61	1.2	12	9	<8	5
3	*B*. *hancei*	WP	4.96	9.9	19	10	13	11
4	*C*. *austroglauca*	TBL	1.50	3.0	23	14	15	9
5	*C*. *japonica*	V	1.57	3.1	9	11	13	8
6	*C*. *orbiculatus*	V	2.06	4.1	11	10	<8	10
7	*C*. *orchioides*	WP	2.28	4.6	16	13	14	8
8	*C*. *prainii*	WP	1.96	3.9	10	10	<8	9
9	*E*. *Burm*	L	3.03	6.1	10	10	10	<8
10	*E*. *daneillii*	TBL	2.52	5.0	18	12	10	9
11	*E*. *fortunei*	V	4.42	8.8	15	13	12	11
12	*E*. *laxiflorus*	TBL	2.68	5.4	<8	<8	<8	10
13	*G*. *morella*	WP	7.38	14.8	17	12	<8	<8
14	*I*. *simonsii*	TBL	3.48	7.0	13	11	11	8
15	*K*. *angustifolia*	TBL	0.99	2.0	<8	8	<8	8
16	*L*. *lancifolia*	TBL	2.28	4.6	<8	<8	<8	<8
17	*M*. *hongheensis*	TBL	3.09	6.2	21	11	13	<8
18	*M*. *salicina*	TBL	2.91	5.8	12	9	8	10
19	*M*. *squamulata*	TBL	1.48	2.9	17	12	12	11
20	*M*. *yunnanensis*	TBL	2.91	5.8	24	12	<8	9
21	*O*. *javanica*	WP	2.65	5.3	<8	8	<8	<8
22	*P*. *edulis*	TBL	4.49	9.0	<8	<8	<8	<8
23	*P*. *molle*	WP	2.99	6.0	11	10	16	12
24	*R*. *japonicus*	WP	5.24	10.5	<8	9	8	<8
25	*S*. *arborescens*	TBL	1.86	3.7	24	13	14	11
26	*S*. *davidii*	TBL	2.54	5.1	<8	8	9	10
27	*S*. *parasitica*	TBL	1.04	2.1	<8	<8	<8	<8
28	*S*. *sinensis*	TBL	6.49	13.0	18	8	13	21
29	*S*. *tamariscina*	WP	4.44	8.9	12	9	11	9
30	*S*. *viridis*	V	2.63	5.3	12	<8	<8	9

^a^ IZD: Inhibition zone diameter (the concentration of the extract at 50 mg/mL); ^b^ Part: The part of plant used for extraction (L: leaves; TBL: tender branches and leaves; WP: whole plant; V: vane); ^c^ Weight: The weight of extract; ^d^ Ratio: The ratio of extract; ^e^ SA: *Staphylococcus aureus* (ATCC 25923, MSSA); ^f^ EC: *Escherichia coli* (ATCC 25922); ^g^ PA: *Pseudomonas aeruginosa *(ATCC 27853); ^h^ CA: *Candida albicans *(ATCC Y0109).

**Table 4:** Comparison of IZDs of the 21 extracts against MRSA and MSSA strains (IZD: mm) ^a^.

No.	Extracts	MSSA	MRSA_ave_ ± SEM (n) ^b^	△(MSSA-MRSA_ave_)
1	*M. yunnanensis*	24	17.5 ± 1.04 (4)	6.5
2	*S. arborescens*	24	18.8 ± 2.06 (4)	5.2
3	*C. austroglauca*	23	18.5 ± 1.85 (4)	4.5
4	*M. hongheensis*	21	15.8 ± 1.65 (4)	5.2
5	*B. hancei*	19	16.0 ± 1.63 (4)	3.0
6	*S. sinensis*	18	14.3 ± 1.31 (4)	3.7
7	*E. daneillii*	18	13.8 ± 1.31 (4)	4.2
8	*M. squamulata*	17	14.5 ± 0.87 (4)	2.5
9	*G. morella*	17	15.7 ± 0.67 (3)	1.3
10	*C. orchioides*	16	17.3 ± 0.95 (4)	−1.3
11	*E. fortunei*	15	15.5 ± 1.55 (4)	−0.5
12	*A. nepalensis*	14	17.0 ± 1.41 (4)	−3.0
13	*I. simonsii*	13	12.8 ± 1.11 (4)	0.2
14	*B. balsamifer*	12	7.0 ± 1.22 (4)	5.0
15	*S. viridis*	12	11.0 ± 1.68 (4)	1.0
16	*S. tamariscina*	12	11.0 ± 1.00 (4)	1.0
17	*M. salicina*	12	9.7 ± 0.33 (3)	2.3
18	*P. molle*	11	15.8 ± 1.38 (4)	−4.8
19	*C. orbiculatus*	11	9.5 ± 0.50 (4)	1.5
20	*E. Burm*	10	13.0 ± 2.08 (3)	−3.0
21	*C. prainii*	10	9.0 ± 1.00 (2)	1.0

^a^ IZD: Inhibition zone diameter (the concentration of the extract at 50 mg/mL); ^b^ Number of MRSA isolates.

**Table 5:** Anti-MRSA activity of the extracts to the zone of inhibition against MSSA ≥10 mm (MIC/MBC: μg/mL).

No.	Extracts and Vancomycin	Activity	MSSA	MRSA 082	MRSA 092	MRSA 189	MRSA 144	MRSA 321
1	*M*. *yunnanensis*	MIC	32	8	32	64	16	32
		MBC	128	64	256	128	64	256
2	*S*. *arborescens*	MIC	64	64	64	64	16	32
		MBC	256	256	256	256	128	128
3	*C*. *austroglauca*	MIC	64	64	64	64	32	16
		MBC	256	128	512	256	128	256
4	*M*. *hongheensis*	MIC	32	128	8	32	16	16
		MBC	128	512	32	128	64	64
5	*B*. *hancei*	MIC	64	64	32	64	32	64
		MBC	128	256	256	128	128	256
6	*E*. *daneillii*	MIC	32	32	32	32	32	64
		MBC	64	256	128	256	256	256
7	*S*. *sinensis*	MIC	32	64	32	32	32	16
		MBC	128	256	256	64	128	64
8	*G*. *morella*	MIC	32	32	64	32	16	32
		MBC	256	256	256	128	64	128
9	*M*. *squamulata*	MIC	64	32	64	32	64	32
		MBC	256	128	256	128	256	128
10	*C*. *orchioides*	MIC	512	256	512	512	512	256
		MBC	1,024	1,024	>2,048	>2,048	512	1024
11	*E*. *fortunei*	MIC	512	512	512	512	512	512
		MBC	>2,048	>2,048	1,024	>2,048	>2,048	>2,048
12	*A*. *nepalensis*	MIC	1024	512	256	512	512	256
		MBC	>2,048	1,024	1,024	>2,048	>2,048	1,024
13	*I*. *simonsii*	MIC	512	512	512	1,024	1,024	1,024
		MBC	1,024	>2,048	>2,048	>2,048	>2,048	>2,048
14	*B*. *balsamifer*	MIC	256	256	256	64	256	128
		MBC	1,024	256	1,024	256	1,024	256
15	*M*. *salicina*	MIC	512	512	1,024	512	512	512
		MBC	1,024	>2,048	>2,048	>2,048	1,024	>2,048
16	*S*. *viridis*	MIC	256	128	64	128	128	128
		MBC	1,024	512	256	512	512	512
17	*S*. *tamariscina*	MIC	512	1,024	1,024	1,024	1,024	1,024
		MBC	1,024	>2,048	>2,048	2,048	>2,048	>2,048
18	*C*. *orbiculatus*	MIC	1,024	1,024	512	512	1,024	512
		MBC	>2,048	>2,048	1,024	1,024	>2,048	1,024
19	*P*. *molle*	MIC	512	256	256	256	256	256
		MBC	>2,048	1,024	1,024	1,024	1,024	1,024
20	*C*. *prainii*	MIC	1,024	1,024	1,024	1,024	1,024	2,048
		MBC	>2,048	>2,048	2,048	>2,048	>2,048	>2,048
21	*E*. *Burm*	MIC	512	512	1,024	1,024	1,024	1,024
		MBC	>2,048	1,024	>2,048	>2,048	>2,048	>2,048
/	Vancomycin	MIC	1	1	1	1	1	1
		MBC	2	2	2	2	2	2

### 2.2. Discussion

A collection of 30 Chinese medicinal plants were evaluated for their *in vitro* antimicrobial effects, especially their anti-MRSA potentials. Most of the plants with anti-infective effects related to skin infections have been indicated in various works of Chinese herbal medicines either by themselves or the different species belonging to the same botanical genus, such as the indication of carbuncle which means a severe abscess or multiple boil in the skin, typically infected with *S*. *bacteria* (Table 1) [6,7,8,9,10,11]. The antimicrobial screening of all the plant species is being reported for the first time to the best of our knowledge, especially the anti-MRSA activities.

The 80% ethanol extracts of the 30 Chinese medicinal plant extracts were initially subjected to the screening of their antimicrobial activities against MSSA and other conventional standard strains of *Escherichia coli* (ATCC 25922), *Pseudomonas aeruginosa *(ATCC 27853) and *Candida albicans *(ATCC Y0109). Their activities as IZDs (mm) at a concentration of 50 mg/mL ranged from <8 to 24, of which 21 extracts showed IZDs ≥10 mm against MSSA (Table 1). The results were in agreement with the literature record of traditional usages (Table 3) [6,7,8,9,10,11]. Judging from the IZD values, the extracts were generally more active against the Gram positive pathogen (MSSA) than against Gram negative (*E*. *coli* and *P*. *aeruginosa*) and fungal pathogens (*C*. *albicans*). The additional permeability barrier caused by the outer lipopolysaccharide layer in Gram negative bacteria makes them more resistant to plant natural products, as has been demonstrated previously [13,14]. Therefore, finding out anti-staphylococcal natural products from plant materials is much easier and is proved in the previous reports [15,16,17,18].

The tested MRSA strains were seven multi-drug resistant clinical isolates (Table 2). They were used for the further assay of their susceptibilities to the 21 extracts (Table 4). It is interesting that these extracts which were active against MSSA also showed more or less activity against MRSA with IZD values (mm) ranging between 9.0 and 18.8 and this has been noted previously [15,16,17,18,19]. The differences were from −3.0 to 6.5 mm in this study (Table 4).

Anti-MRSA potency of MICs/MBCs (μg/mL) of the 21 extracts was determined as 8-2,048/32->2,048 (Table 5). Comparison of the values of IZD and MIC for a same extract in Table 4 and Table 5, they were not always positively correlated. As different sample concentrations will produce different IZDs, the real inhibitory potency of a sample should be judged by its MIC. This should be noted for future studies.

From the phytochemical constitution of the 30 plant materials listed in Table 1, it is found that most of the active extracts contained tannins, (poly)phenols (including flavonoids, lignans and coumarins), terpenoids or alkaloids which have been reported [18,20,21,22,23,24,25].

The phytochemicals, including polyphenols and antimicrobial activity of *Mallotus* species have been reviewed [26,27]. Rottlerin from *M*. *philippinensis* exhibited potent bactericidal activity with an MBC value of 3.12–6.25 μg/mL against several clinical *H. pylori* isolates [28]. *Garcinia* genus is rich in caged xanthones [29]. Morellin isolated from gamboge (*G*. *hanburyi*,tenghuang in Chinese) was reported as an antibiotic principle in 1954 [30]. The anti-MRSA activity of morellin and other caged xanthones from Gamboge, *G*. *morella* and other *Garcinia* species have also reported [31,32]. Two antifungal compounds ulopterol (a coumarin) and a quinolone alkaloid 4-Methoxy-l-methyl-3-(2'-S-hydroxy-3'-ene-butyl)-2-quinolone were isolated from *Skimmia laureola*, a Pakistani medicinal plant [33], which were similar to our results of the inhibition of *S. arborescens* against *C. albicans* (Table 3). However, no active antimicrobial compounds from the other plant materials have been isolated up to now, especially the anti-MRSA compounds from the most active extracts of *S*. *sinensis*, *E*. *daneillii*, *M*. *squamulata*, and *B*. *hancei*, with MICs at 8–64 μg/mL. We are continuing our search for the corresponding phytochemicals from these plants and their further systematic anti-MRSA properties.

## 3. Experimental

### 3.1. Plant Materials

The selected 30 Chinese medicinal plant samples were collected from the tropical mountain forests of southeastern Yunnan Province of China, at altitudes of 1,500–3,074 m in June 2010. They were identiﬁed by Y.M. Shui at the Kunming Institute of Botany (KIB); the Chinese Academy of Sciences. Voucher specimens are preserved at the herbarium of KIB [34]. The names of species/family (specimen No.) were as the following: *Alnus nepalensis* D. Don./Betulaceae (KUN 35); *Blumea balsamifer* (Linn.) D.C./Asteraceae (KUN 21); *Brandisia hancei* Hook. f./Scrophulariaceae (YFS 6); *Carex prainii* C.B. Clarke/Cyperaceae (KUN 11133); *Cayratiy japonica *(Thunb) Gngacp.var.*japonica*/Vitaceae (YFS 531); *Celastrus orbiculatus* Thunb./Celastraceae (KUN 110); *Curculigo orchioides *Gaertn./Hypoxidaceae (KUN 1151); *Cyclobalanopsis austroglauca *Y.T. Chang/Fagaceae (SWFC 851222); *Embelia* Burm f./Myrsinaceae (KUN355); *Euonymusfortunei* (Turcz.); Hand. Mazz./Celastraceae (YUKU (s.n)); *Euonymus laxiflorus* Charmp.ex.Benth./Celastraceae (YCP 851027); *Evodia daneillii *(Benn) Hemsl./Rutaceae (YFS 663); *Garcinia morella* Desr./Clusiaceae (YCP 851757); *Illicium simonsii* Maxim./Il1iciaceae (IBSC 125); *Kadsura* angustifolia A.C. Smith/Schisandraceae (KUN 4480); *Litsea lancifolia* (Roxb. ex. Nees); Benth. et. Hook. f. ex. F./Lauraceae (KUN 23); *Machilus salicina* Hance./Lauraceae (YUKU 1535); *Mallotusyunnanensis *Pax et. Hoffm./Euphorbiaceae (YFS 1144); *Manglietia hongheensis *Y.m Shui et. W.H. Chen./Magnoliaceae (KUN 262); *Meliosma squamulata* Hance./Lauraceae (KUN 2411); *Oenanthe javanica* (Bl.)DC./Umbellifera (KUN 214); *Polygonummolle* D. Don./Polygonaceae (KUN 367); *Pyrularia edulis* (Wall.) A.D.C./Santalaceae (KUN 60002100); *Ranunculus japonicus* Thumb./Ranunculaceae (KUN 2398); *Schima sinensis* (Hemsl. et. Wils) Airy-shaw./Theaceae (KUN158); *Schisandra viridis* A.c.Smith./Schisandraceae (YFS); *Scurrula parasitica *Linn.var. *parasitica*./Loranthaceae (YFS 327); *Selaginella tamariscina* (Seauv.) Spring./Selaginellaceae (YCP 85937); *Skimmia arborescens* Anders./Rutaceae (PE 100260); *Sophora davidii *(Franch.) Skeels./Leguminosae (YCP 851049). The traditional indications and phytochemical constitutions were listed in Table 1.

### 3.2. Microbial Strains and Culture Media

Standard bacterial and fungal strains, *i.e*., *Staphylococcus aureus* (ATCC 25923, MSSA), *Escherichia coli* (ATCC 25922), *Pseudomonas aeruginosa *(ATCC 27853) and *Candida albicans *(ATCC Y0109) were provided by the National Institute for the Control of Pharmaceutical and Biological Products (NICPBP, China). Clinical MDR MRSA strains of MRSA 8, MRSA 82, MRSA 92, MRSA 123, MRSA 144, MRSA 189 and MRSA 321 were clinical isolates from infectious samples of critically ill patients in Kunming General Hospital (KGH). Pathogen puriﬁcation and identiﬁcation (including colonial morphology, Gram staining and coagulase testing) were conducted in our clinical microbiology laboratory and further conﬁrmed by standard cefoxitin disk diffusion test following the Clinical and Laboratory Standards Institute (CLSI) guidelines [15,35,36]. ATCC 25923 was used as the control strain. Vancomycin (Eli Lilly Japan K.K., Seishin Laboratories) was used as a control anti-MRSA agent. Standard Mueller-Hinton agar and broth (MHA and MHB) and Sabouraud agar (Tianhe Microbial Agents Co., Hangzhou, China) were used as the bacterial and fungal culture media, respectively. The antibacterial agents-susceptibility testing results of MRSA strains were shown in Table 2.

### 3.3. Extract Preparation

The 30 samples of the air-dried and ground plant materials (50 g) were macerated with 80% ethanol (500 mL) for 7 days, ﬁltered and the mare was further macerated twice with the same solvent overnight and ﬁltered after being sonicated for 30 min. The ﬁltrates were combined and the solvent was evaporated at 40 °C in vacuum to afford each of the plant extract (Table 3).

### 3.4. Antimicrobial Screening

The ethanol extracts of the 30 plants were primarily subjected to susceptibility screening against standard microbial strains according to the agar diffusion method on MHA (for the bacteria) or SA (for *C*. *albicans*) plates. The sample extracts (50 mg/mL in dimethyl sulfoxide) were pipetted into 6 mm (in diameter) holes punched in the agar of prepared agar plates, plating with inoculums of 1.5 × 10^8^ CFU/mL for bacteria and 5 × 10^5^ CFU/mL for *C*. *albicans* in advance, respectively and incubated at 35 °C (for *C*. *albicans* at 28 °C) for 24 h, measured and recorded the IZDs [15,16,17,37]. The solvent value was deducted accordingly to get ﬁnal results of activity. All experiments were carried out in triplicate. The test results were interpreted based on IZD as <10 mm for the resistance, =10 mm for the mildly susceptible, 11–15 mm for moderately susceptible, ≥16 mm for the highly susceptible (Table 2). The 21 extracts with IZDs ≥10 mm against MSSA were further subjected to the assay of their IZDs against MRSA strains (Table 3).

### 3.5. MICs and MBCs Assaying

The extracts with IZDs ≥10 mm against MSSA were further subjected to the assay of their IZDs and minimal inhibitory concentrations and minimal bactericidal concentrations (MICs/MBCs) against MRSA strains by serial dilution method according to the procedures reported previously [15,16,17,38,39]. Briefly, MICs/MBCs were determined by the standardized broth (using MHB) microdilution techniques with starting inoculums of 5 × 10^5^ CFU/mL for the bacteria according to CLSI (formally NCCLS) guidelines and incubated at 35 °C for 24 h. For the MBCs assaying, 0.1 mL aliquots from drug dilution wells with visual growth inhibition were plated onto MHA media. The lowest drug concentration that yielded three or fewer microorganism colonies was recorded as the MBC. They were determined in triplicate, with concentrations ranging up to 2048 μg/mL for all the extracts (Table 5).

## 4. Conclusions

The screening of *in vitro* antimicrobial activity of the ethanol extracts from 30 Chinese medicinal plants led to the confirmation of 21 extracts displaying both anti-MSSA and MRSA effects with various levels of potency which were in good agreement with their TCM indications of skin infections and modern phytochemical constituents, with *M*. *yunnanensis* and *S*. *arborescens* extracts being the most active against MRSA. The combination of TCM indications and phytochemical profiles is a promising approach to the search of anti-MRSA plant natural products.
